# MANDIBULAR CONDYLAR PROCESS REMODELING IN RATS WITH DIFFERENT BITE-ALTERING DEVICES

**DOI:** 10.22203/eCM.v045a04

**Published:** 2023-02-14

**Authors:** W. Li, S. Trbojevic, J.B. Pineda-Farias, X. Liu, M.S. Gold, A.J. Almarza

**Affiliations:** 1 Department of Oral and Craniofacial Sciences, School of Dental Medicine, University of Pittsburgh, Pittsburgh, PA 15261, USA; 2 Center for Craniofacial Regeneration, University of Pittsburgh, Pittsburgh, PA, 15261, USA; 3 Department of Bioengineering, Swanson School of Engineering, University of Pittsburgh, Pittsburgh, PA 15261, USA; 4 Department of Neurobiology, School of Medicine, University of Pittsburgh, Pittsburgh, PA 15261, USA; 5 McGowan Institute of Regenerative Medicine, University of Pittsburgh, Pittsburgh, PA, 15261, USA

**Keywords:** Temporomandibular joint, temporomandibular joint disorder, occlusal splint, degenerative joint disease, TMJ degeneration models

## Abstract

The objective was to compare different dental splint models and materials for inducing abnormal loading on the gross morphology and histological appearance of the mandibular condylar processes of Sprague Dawley rats. Three different types of dental splints (resin molar, aluminum incisor, stainless-steel incisor) were placed unilaterally to induce occlusal perturbation for 4 weeks. At that time, mandibular condylar processes were assessed by gross appearance and histology. Quantitative measurements were also conducted on the hematoxylin & eosin images for condyle shape. The results showed that although the condylar cartilage was affected by all splint types, the resin molar splint was associated with the most extensive mandibular condylar process remodeling, which was primarily a slant (skewness) of the lateral aspect of the condylar process. Additionally, quantitative measurements on the histological specimens demonstrated that the split and tilt angle of the left (ipsilateral) condylar processes in the resin molar group (124.8 ± 12.7° and 104.1 ± 12.7°, respectively) increased significantly (*p* < 0.05) when compared to right (contralateral) condylar processes (104.7 ± 5.8°and 91.6 ± 4.4°, respectively). However, no changes were noted on the thickness of the fibrocartilage layer at medial, central, and lateral regions of the condylar process. Another major finding is the high variability of morphology of the naïve animals. Future studies will assess the impact of longer durations of splinting, age, and sex on the remodeling of the mandibular condylar process, allowing for the development of diagnostics and therapies.

## Introduction

As a load-bearing structure, the TMJ is under repetitive functional loading from jaw movements and mastication (Boyd *et al.*, 1990; Brehnan *et al.*, 1981). Thus, the homeostasis of anabolic and catabolic events involving the fibrocartilage and subchondral bone in the mandibular condyle is crucial to the normal function of the TMJ. A disruption of this balance could result in DJD of the TMJ, which might be associated with pain. The etiology of painful DJD is still not understood, as it is multi-factorial across a heterogeneous patient population (Dworkin *et al*., 1990; Smith *et al*., 2011; Von Korff *et al*., 1988). Tooth loss, periodontal diseases, traumas, or dental treatments such as orthodontic and prosthodontic treatments can potentially cause malocclusion or occlusion disharmony, which was believed to be the risk factor for painful DJD (Cao, 2022; Michelotti and Iodice, 2010). Pathological changes such as degeneration of the articular mandibular cartilage are seen on 11 % of individuals with TMJ disorders (Mejersjö and Hollender, 1984), which can then lead to a cascade of problems resulting from functional and morphological changes in the joint (Zarb and Carlsson, 1999). The pathological process of joint degeneration includes irreparable abrasion of articular cartilage and thickening and remodeling of underlying bone (Zarb and Carlsson, 1999). However, reviews have pointed out that there was still not enough evidence to indicate a clear relationship between occlusal disharmony and masticatory function as orthodontic treatments, when done properly, were reported to reduce the risk of TMD/DJD (Christensen and Rassouli, 1995; Clark *et al.*, 1999; Greene and Laskin, 1988).

This unknown and complex etiology makes it difficult to develop animal models of the DJD (National Academies of Sciences and Medicine, 2020). A good model of DJD should not require surgical access into the joint and cause secondary inflammation that would confound results. Thus, both the use of irritants and abnormal loading models have been pursued to cause non-invasive joint damage. However, irritants cause acute hypersensitivity and rapid joint damage (Clemente *et al.*, 2004; Fischer *et al.*, 2008; Gameiro *et al.*, 2005; Gameiro *et al.*, 2006; Hutchins *et al.*, 2000; Iwata *et al.*, 1999; Ren, 1999; Roveroni *et al.*, 2001; Shiojiri *et al.*, 2002; Zhou *et al.*, 1999), which may not be representative of the presentation of TMJ patients that is usually associated as a chronic pain disease. Thus, abnormal loading models (Almarza *et al.*, 2011; Guo *et al.*, 2020; Henderson *et al.*, 2015a; Henderson *et al.*, 2015b; Honzawa *et al.*, 1988; Li *et al.*, 2010; Wang *et al.*, 2018a; Xu *et al.*, 2019) is a physiologically relevant approach to drive relatively mild degenerative changes in the joint, or OA, over a longer time span and potentially with slowly evolving hypersensitivity.

An increasing number of studies have shown that unbalanced loading on TMJs from bite altering splints/blocks can cause the remodeling of the MCC and the subchondral bone (Abdalla *et al.*, 2019; Almarza *et al.*, 2018; Buchner, 1982; Ghafari and Degroote, 1986; Henderson *et al.*, 2015a; Kuang *et al.*, 2019; Long *et al.*, 2019; Mao *et al.*, 1998; Wang *et al.*, 2019; Zhang *et al.*, 2015; Zhang *et al.*, 2018). However, there is inconsistency in the literature as to the extent and nature of the remodeling observed following abnormal loading of the joint. This is likely due to differences in splinting techniques, as they can be placed on the incisor teeth (Farias-Neto *et al.*, 2012; Ghafari and Degroote, 1986; Guo *et al.*, 2020; Sá *et al.*, 2017; Wang *et al.*, 2018b; Watanabe *et al.*, 2008) or the molar teeth, and if on the molar teeth the splints can be unilateral (Abdalla *et al.*, 2019; Gazit *et al.*, 1987; Mao *et al.*, 1998) or bilateral (Buchner, 1982; Li *et al.*, 2010; Long *et al.*, 2019). Furthermore, the splints can be placed on the maxillary (Gazit *et al.*, 1987; Li *et al.*, 2010; Long *et al.*, 2019; Mao *et al.*, 1998) or mandibular molar teeth (Abdalla *et al.*, 2019; El-Bialy *et al.*, 2015). Moreover, the lack of reliable quantitative parameters to describe the remodeling has limited the ability to make direct comparisons between models.

Marked changes to the condylar process were reported in all studies in which unilateral molar splints were used (Abdalla *et al.*, 2019; Gazit *et al.*, 1987; Mao *et al.*, 1998). However, there was no assessment of the joint contralateral to the splint in these studies, despite the possibility that bilateral changes that might be more reflective of the human condition. As for incisor splints, studies also have shown an impact on condylar process remodeling (Farias-Neto *et al.*, 2012; Ghafari and Degroote, 1986; Guo *et al.*, 2020; Sá *et al.*, 2017; Wang *et al.*, 2018b; Watanabe *et al.*, 2008), but the effects observed were the same on both condylar processes. Therefore, the objective of this study was to compare the impact between different splint methods (incisor and molar) and materials (resin, aluminum, stainless steel) for altering the bite on the gross and histological appearance of the mandibular condylar processes of Sprague Dawley rats.

## Materials and Methods

### Animals

A total of 28 female Sprague Dawley rats were used in this study. The rats were purchased (Envigo, Indianapolis, USA) at the age of around 11 weeks and were housed in micro-isolator boxes in groups of two in an AAALAC approved animal facility with tight control over temperature (~ 23 °C) and humidity (50–60 %), in a room on a 12:12 light cycle (lights on at 7:00 a.m.). Standard rat chow and tap water were provided *ad libitum*. The facility is managed by the Division of Laboratory Animal Resources in the University of Pittsburgh, and all procedures involving animals were approved by the institutional animal care and use committee and performed in accordance with the policy on humane care and use of laboratory animals and the NIH guide for the care and use of laboratory animals (NIH Publications No. 8023, revised 1978).

After their arrival at the facility, rats were allowed to acclimate for at least 2 d before any procedures were performed. All the rats were randomly assigned to 1 of 4 groups, using block procedure: RM splint group, ALI splint group, SSI splint group, and NC group at 12 weeks old (*n* = 7 for each group). This was an exploratory study to investigate the outcome of different splints in inducing DJD in rats in which what variability to expect from both naïve and the different splinting groups was not known. Thus, *n* = 7 gave sufficient samples of each remodeling phenotype observed for confidence that changes between splinting and naïve were recognised. However, future studies will need to assess at least 5 if not 10 animals to ensure repeatability. The rats in RM, ALI and SSI group received corresponding bite-altering splints (as described below) at the age of around 12 weeks, while no procedures were performed on the NC group.

Sprague Dawley rats were purchased at 11 weeks old, and the splints were placed at 12 weeks old. According to a previous study (Sengupta, 2013), rats enter their adulthood at the 8th week post-natal and a 12-week-old Sprague Dawley rat is considered an adult. Undeniably, using > 20-month-old rats corresponds to middle-aged women in humans and would be ideal for studying the development of DJD. In a pilot study, using retired female breeding 10–12 month-old rats, similar outcomes were seen. Therefore, it was decided that 12-week-old rats would be used since their growth was already plateauing and there were no increased costs associated with aging the rats. Future studies on the remodeling of the condylar process on abnormal loading models should look at the impact of age, specifically using at least 20-month-old rats.

**Fig. 1.**
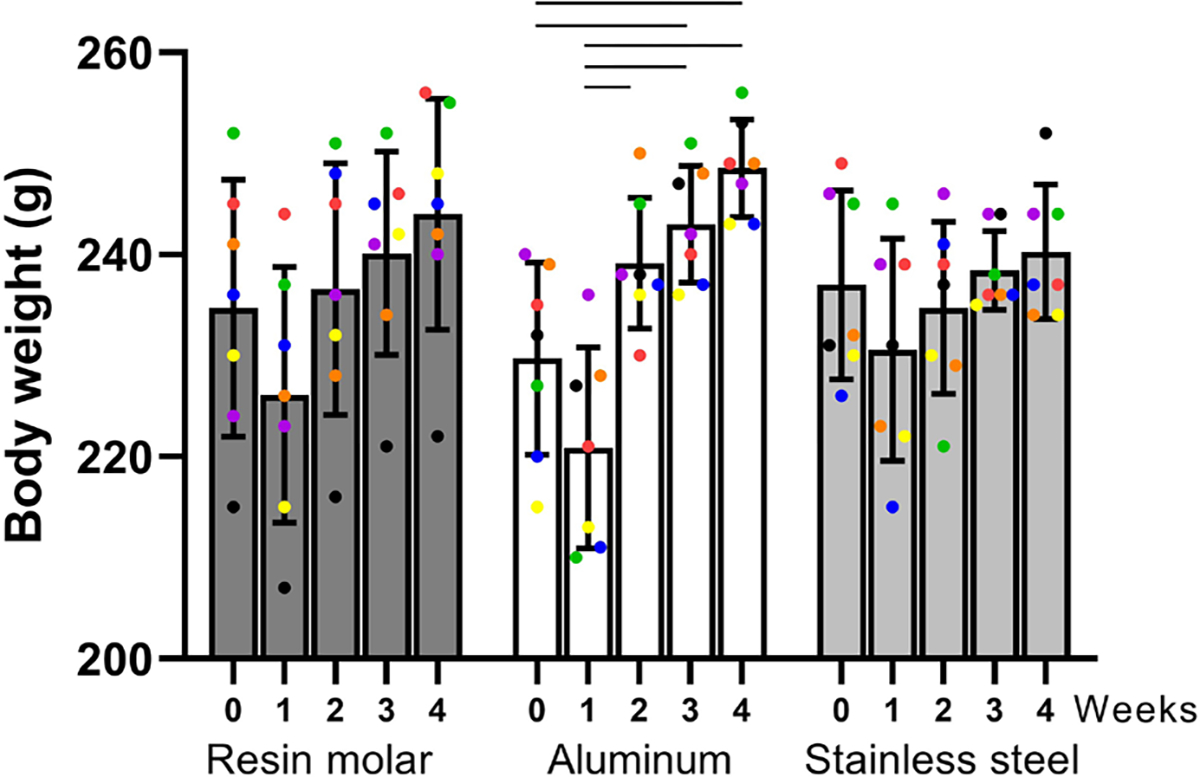
Rat body weights. Each color-coded dot represents one rat in each group. Significance (*p* < 0.05) were seen within the aluminum group but not in the RM nor SSI group.

The body weight of each rat was monitored and recorded weekly. There was no evidence of severe malnutrition (Fig. 1); therefore, no rats were excluded from the study. At the end of the 4-week splinting period, all the rats were euthanized. As the objective of this project was to develop/adapt different splint methods, gross morphology and histology were not blinded to the groups, because it was not known what differences, if any, to expect. Confounders, such as animal/cage location or the order of treatments and measurements, were not controlled.

### Splint placement

**Fig. 2.**
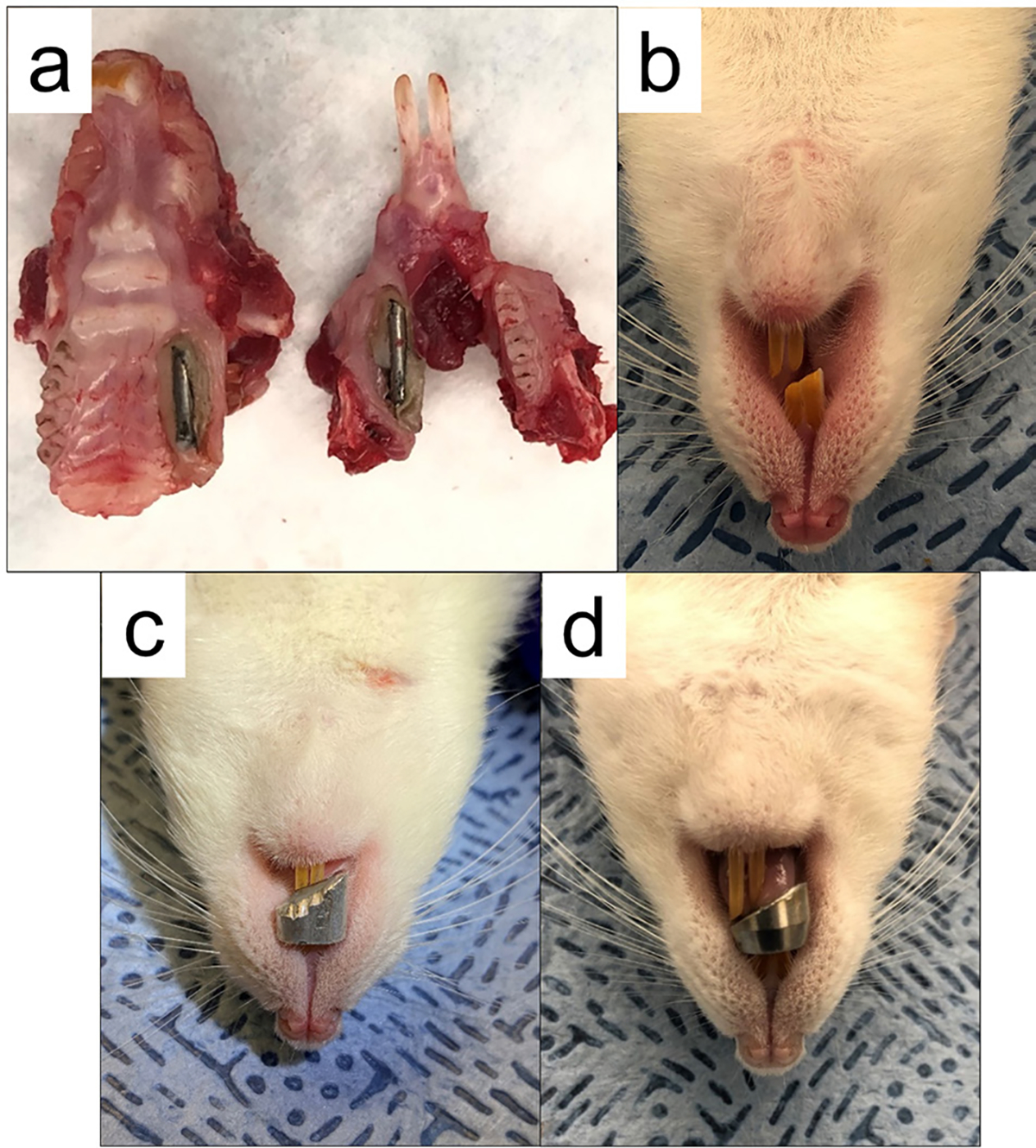
Examples of different bite-altering splints. (**a**) RM splint. (**b**) Uneven wear of the incisor teeth. (**c**) ALI splint. (**d**) SSI splint.

For the RM splint, pilot studies showed that placing a 1 mm resin splint was not sufficient to induce condylar remodeling consistently, and that such a thin layer of resin was easily ground down. Therefore, in this study, 1 mm resin splints were placed unilaterally on both the upper and lower molar teeth of the rats. Because of the restricted space in the rat mouth, it was not possible to put a matrix band around the molar teeth and build up the splint with resin composite. Therefore, flowable resin was used to build up the splint layer by layer. The initial layers were critical, because the resin got into the pits, grooves, and undercuts of the molar teeth to provide a mechanical anchorage, resulting in a good splint retention. Prior to splinting, rats were anesthetized with an intraperitoneal injection of a mixture consisting of 0.55 g/mL ketamine, 0.275 g/mL xylazine, and 0.11 g/mL acepromazine, administered at a volume of 1 mL/kg. When rats were areflexive to tail pinch, they were positioned on their back with their mouth held open by a combination of a rubber band and modified mouth prop. All the left upper and lower molar teeth were cleaned with water, etched with 38 % phosphoric acid (Etch-Rite etching gel, Pulpdent, USA) for 30 s, rinsed with water, dried with cotton, and primed with Adper Single Bond 2 (3M ESPE, Germany), followed by a UV light cure for 10 s. The splint was then built on the prepared teeth using Beautifil Flow Plus F00/F03 resin (SHOFU Dental Corporation, USA). In addition, due to its low strength, the resin splint was reinforced using a 1 mm diameter round stainless-steel pin (Fig. 2a). The resin was cured using UV light for 20 s. The thickness of each splint was approximately 1 mm. After the splint placement, rats were anesthetized using 4 % isoflurane delivered *via* the nose, followed by a brief mouth checkup, at least once a week for 4 weeks, to ensure the resin splints were still in place.

For the case of incisor splints, pilot studies with the molar splint showed that the rats develop slanted incisor teeth – indicating a lateral shift of the mandible. This finding led to an investigation of slanted incisor splints, which have the added benefit of loading each condylar process differently. For the ALI splint group and the SSI splint group, the rats were sedated using 4 % isoflurane delivered *via* the nose. When rats were areflexive to tail pinch, they were positioned on their back with their mouth held open with a combination of rubber band and modified mouth prop. The upper incisor teeth were cleaned with water and dried with cotton. Then a modified slanted rope sleeve made from either aluminum (1DLD7A, Dayton Electric Mfg, USA) or stainless steel (ST24–4, Loos & Co Inc., USA) was attached to the upper incisor teeth using zinc oxide Eugneol cement (Prevest Denpro, India) (Fig. 2b,c). After the splint placement, the rats were visually checked to make sure the splints were still in place at least once a week for 4 weeks.

### Sample recovery, processing, and histology

At the end of the 4-week splint period, all the rats were euthanized using CO_2_ followed by cervical dislocation. Their left and right mandibular condylar processes and discs were removed. The condylar processes were then fixed using 15 mL/each (approximately 15 times of the sample size) of 4 % paraformaldehyde at 4 °C for 6 h, followed by decalcification in 15 mL/each of Immunocal (StatLab, USA) at 4 °C for 48 h. Gross images were taken from the anterior and lateral side of the condylar processes using a Leica M165FC microscope (Model: MDG41 with DFC 450C. Leica, Germany) under brightfield illumination. The condylar processes were then dehydrated using 70 % ethanol at 4 °C overnight and embedded in paraffin wax. To minimize the variation caused by the selection of slides at different depth, all embedded specimens were processed and trimmed using the same protocol. Specimens were placed on the microtome anterior-posteriorly with the anterior end facing the blade. They were then trimmed until the condylar process was in sight. Consecutive sections were collected at the thickness of 8 μm and 14 μm on to microscope slides, which were then dried on a hot plate at 42 °C overnight and stored in slide boxes at room temperature.

For histology, slides from different rats were chosen at similar depth into the condylar process, which was at around the anterior one third of the condylar process. After deparaffinization, 8 μm slides were stained with H&E, while 14 μm slides were stained in toluidine blue. Images were taken using a Nikon Eclipse microscope (Model: TE2000-E with DS-Fi3 camera. Nikon, Japan) under the brightfield illumination. Images were obtained of both right and left condylar processes. All images of right condylar processes were flipped horizontally for easier comparison.

### Image processing

Images of H&E stained slides were further processed using ImageJ. Split and tilt angles were determined as illustrated in Fig. 6 based on the landmarks of split line, angle divider, and base line. In short, firstly, the medial and lateral borders of the ramus were marked, of which the angle divider was determined (Fig. 5a). Then, the base line was drawn by connecting the medial and lateral convex points below the contour of the condylar process. Next, a line (x) was drawn parallel to the base line and tangential to the surface of the condylar process. The split line was determined by connecting the tangent point and the midpoint of the base line. Finally, the split and base lines dictated the split angle, while the angle divider and the base line dictated the tilt angle. In order to measure the thickness at different regions of the condylar process (Fig. 5b), a central line was drawn from the split line. Then the lateral and medial line were determined by dividing the angles between the central line and the base line on the medial and lateral aspect respectively. Cartilage thicknesses of the medial, central, and lateral regions were measured.

### Statistical analysis

All the weight, angle, and thickness data were reported as mean ± standard deviation (*n* = 7 for each group, passed the Kolmogorov-Smirnov test of normality and Levene’s test of equal variance), over which one-way ANOVA tests were performed followed by the *post-hoc* Tukey’s HSD. All statistical analysis were performed using GraphPad Prism 8. *p* < 0.05 was considered significant. This was an exploratory study to investigate the outcome of different splints in inducing DJD in rats in which it was not known what variability to expect from both naïve and the different splinting groups. Thus, *n* = 7 gave sufficient samples for each remodeling phenotype observed to be confident that differences between splinting and naïve were real. However, further studies will be required to assess whether at least 5, if not 10 animals, are needed to ensure repeatability of results.

## Results

### Rats and splints

In the group with aluminum splints, there seemed to be a decrease in weight from week 0 to week 1, which bounced back from week 1 to week 2 (*p* < 0.05). Similar trends were seen in other groups, but the differences were not statistically significant (Fig. 1). All splints were retained for 4 weeks. The wear pattern on the splints varied as a function of splint placement and material. Some of the RM splints showed minor wear of the resin holding the metal pin, but the metal pins were still intact (Fig. 2a). Uneven wear of both upper and lower incisor teeth of rats in the RM group was seen (Fig. 2b), likely indicating an abnormal trajectory of the mandible while chewing. All the ALI splints showed signs of wear (Fig. 2c), while at the macroscopic level, the SSI splints remained fully intact (Fig. 2d). It is worth pointing out that all the rats in the SSI group developed slanted lower incisor teeth and were offset from the midline. Similar changes were observed in the rats in the ALI group (6 of 7), but in these, the slant of the incisor teeth was less severe, and there was no detectable shift in the jaw. Gross changes to incisor teeth and jaws of the RM splint group were detected in 6 out of 7 rats. In these, the slant of the incisor teeth was more severe than that of the steel incisor splint group. There were similar changes to the jaw angle.

### Gross morphology of condylar processes

**Fig. 3.**
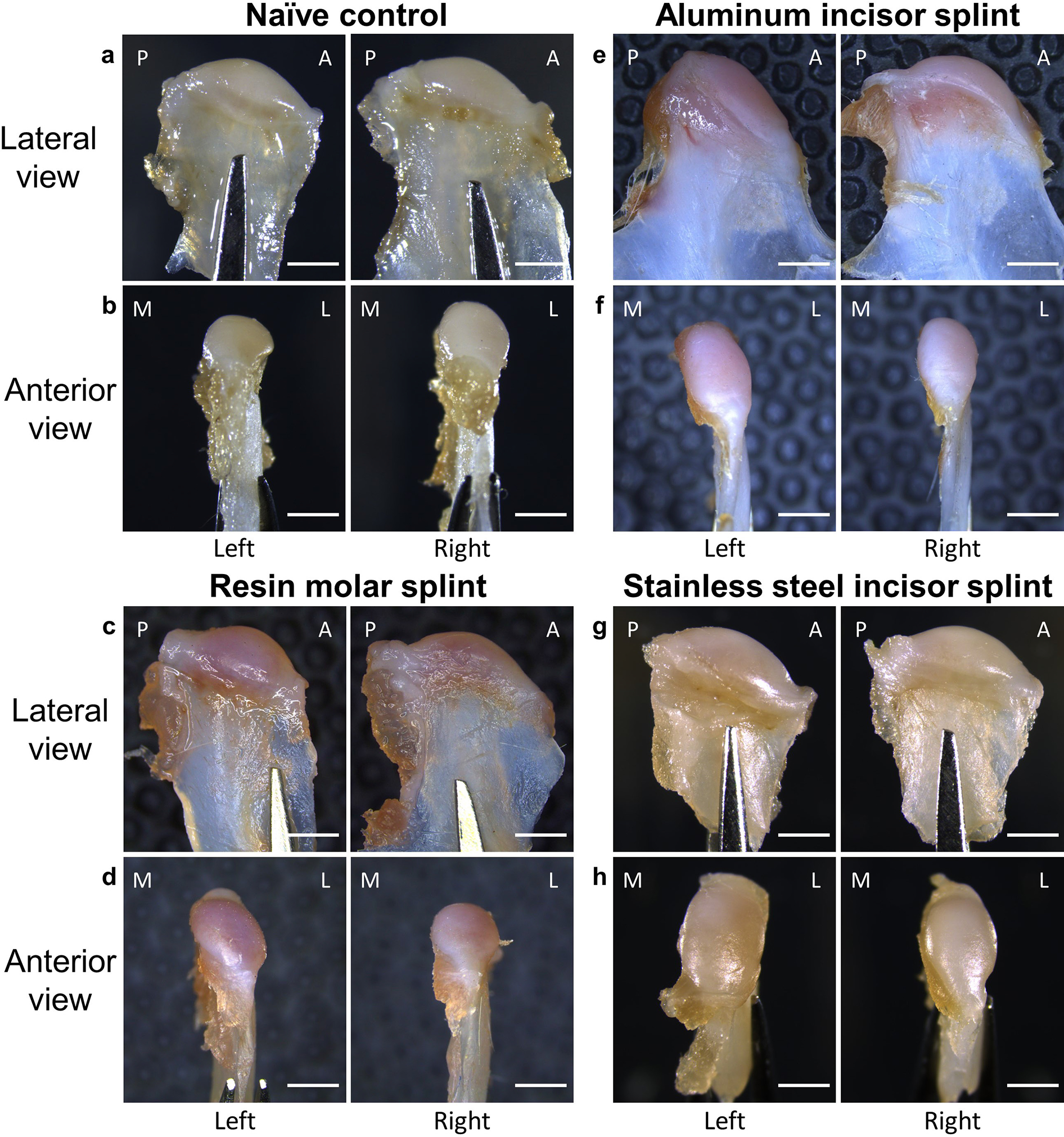
Gross appearance of the condylar processes in different groups. (**a**,**c**,**e**,**g**) Lateral view of left and right condylar processes of the NC, RM, ALI, and stainless-steel groups respectively. (**b**,**d**,**f**,**h**) Anterior view of left and right condylar processes of the NC, RM, ALI, and stainless-steel groups respectively. All right condylar processes were flipped horizontally. A: anterior, P: posterior, M: medial, L: lateral. Scale bar = 500μm.

Analysis of the TMJ tissue sections started with an assessment of the gross appearance of the mandibular condylar processes using a stereomicroscope. The condylar processes of the NC rats looked very similar to their opposing counterparts (left *versus* right) in both lateral and anterior views (Fig. 3a, b). The area of the lateral surface of the left condylar process (Fig. 3c) in the RM group enlarged noticeably from the lateral view compared to the right condylar process. The same location on the left condylar process is flattened, the anterior view compared to the right condylar process and the left condylar process in NC rats, resulting in a slanted condylar process (Fig. 3d). In contrast, the right condylar process in the RM group remained structurally intact with no obvious differences from the morphology of the right condylar process in NC rats (Fig. 3c,d).

In the ALI group, besides the slightly enlarged lateral surface, the left condylar process inclined more towards the anterior when observed from the lateral view (Fig. 3e,f). A similar inclination was observed in the right condylar process of the ALI group but had a smaller lateral surface than the right-hand lateral view (Fig. 3e), which was confirmed by the seemingly missing lateral portion and decreased medial-lateral width of the right-hand condylar process when viewing from the anterior (Fig. 3f).

As expected, changes to the SSI group were more extensive than for the ALI group. In this group, the lateral surface of the right condylar process seemed to be enlarged in the lateral view (Fig. 3g), but instead of becoming flattened, it seemed bulged in the anterior view (Fig. 3h). The overall shape of the right condylar process was similar to that of naïve rats from the lateral view (Fig. 3g), but had an arched top compared to the NC when viewing from the anterior (Fig. 3h).

### H&E staining of condylar processes

Since most of the changes on the condylar process were observed on the lateral surface, coronal sections were used to capture the changes on the lateral surface of the condylar processes.

**Fig. 4.**
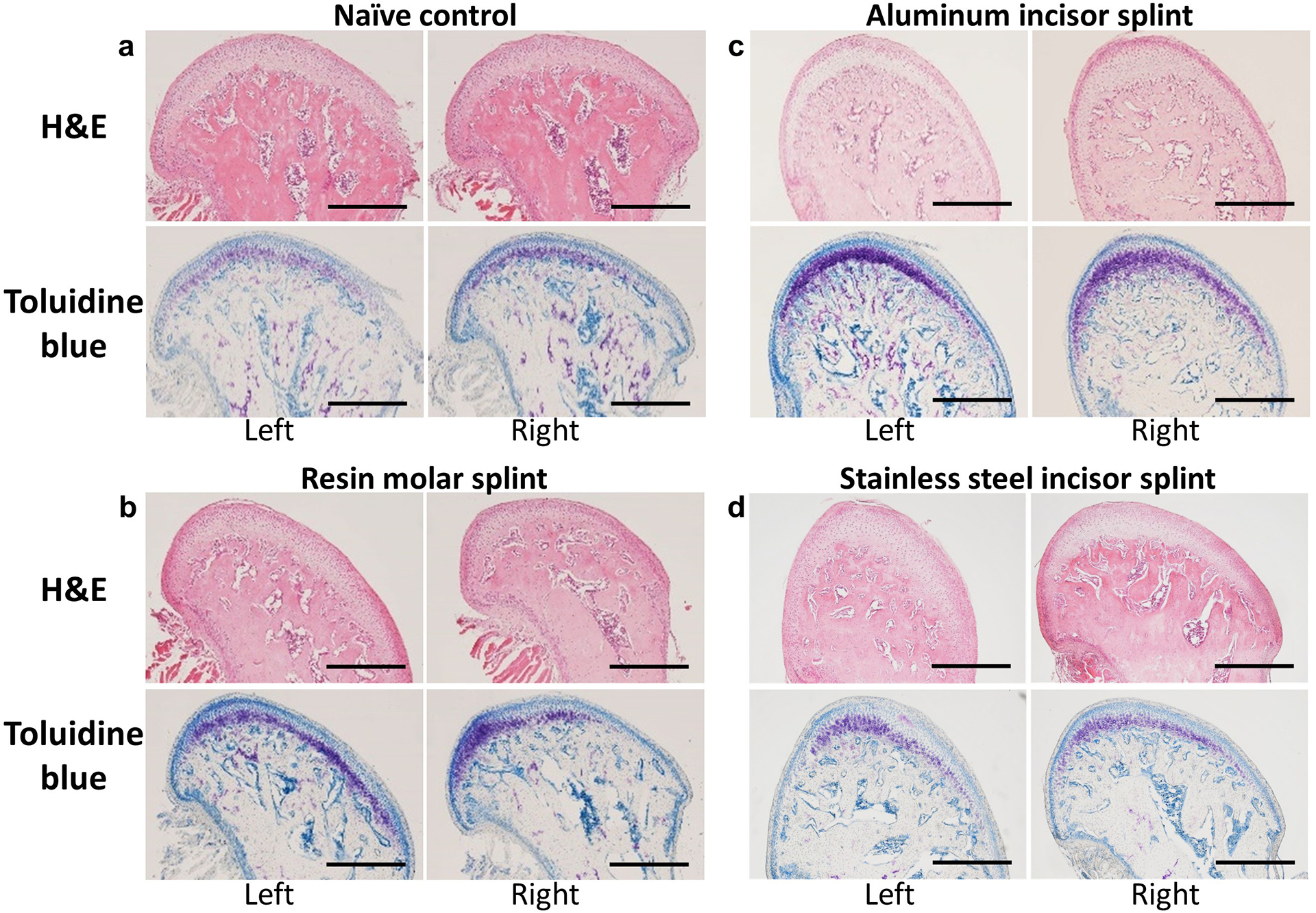
Histology of coronally sectioned condyles. Representative H&E and toluidine blue staining images for left and right condyles of the NC group (**a**), RM group (**b**), ALI group (**c**), and SSI group (**d**). All right condyles were flipped horizontally. Scale bar: 500 μm.

Consistent with what we found in the gross appearance of the condylar process, the lateral surface of the left condylar processes of most rats (6 out of 7) in the RM group were flattened (Fig. 4b) and seemed to be slanting towards the lateral side when compared to NCs (Fig. 4a). The right condylar processes from most rats (6 out of 7) in the RM group (Fig. 4b) were similar to those of the NC group, except for one rat, whose right condylar process was so severely deformed that the normal layers of the fibrocartilage were completely disrupted, as was the articular surface. Both the left and right condylar processes from that rat had numerous circular or elliptic voids in the subchondral bone replacing the normal intertrabecular space.

For the ALI group, the main changes associated with the condylar processes were that their lateral portion was missing, resulting in a sharp and arched top (Fig. 4c). It is worth mentioning that the impact of the ALI splint on condylar process morphology and histology varied dramatically between rats, thus the aforementioned changes were seen in 4 out of 7 rats and might happen to both, one or neither

The lateral surface of the left condylar process also appeared to be the site of the primary impact of the SSI splint, where a slightly slanted lateral surface can be seen (4 out of 7) (Fig. 4d), while no significant changes were noted on the right condylar process (6 out of 7) (Fig. 4d). In the same way as for the ALI group, the histology outcome for the SSI group varied between rats. For example, in two rats, the fibrocartilage layer in the lateral portion of the left condylar process was decreased and the stratified layers were disrupted, while the right condylar process of one rat appeared to be smaller than others.

### Toluidine blue staining of condylar processes

To further investigate how different splinting methods and materials affected the distribution of GAG, toluidine blue staining was performed. The GAG distribution followed a similar pattern to the overall shape of the condylar process. In the NC group, GAG was evenly distributed in the hypertrophic layer from medial to lateral, and both condylar processes shared similar pattern (Fig. 4a). In the RM splint group, the distribution of GAG was very different between left and right condylar processes (Fig. 4b). In the left condylar process, the GAG was evenly distributed in the hypertrophic layer from medial to lateral (Fig. 4b), while in the right condylar process, the GAG was disrupted at the lateral portion (Fig. 4b).

It can be observed in the ALI group and SSI group that the relative thickness of GAG layer was increased (Fig. 4c,d). It is worth pointing out that the GAG layer and distribution in the left condylar process of one splinted rat did not appear to be continuous, a pattern not observed in any condylar processes of NC rats. In contrast, there was no lateral GAG staining observed in the condylar processes from all but two rats in the stainless-steel splint group. In these two rats there appeared to be more GAG staining in either the left or the right condylar process. Furthermore, the proportional thickness of the GAG layer of some condylar processes was decreased.

### Measurement of landmarks

**Fig. 5.**
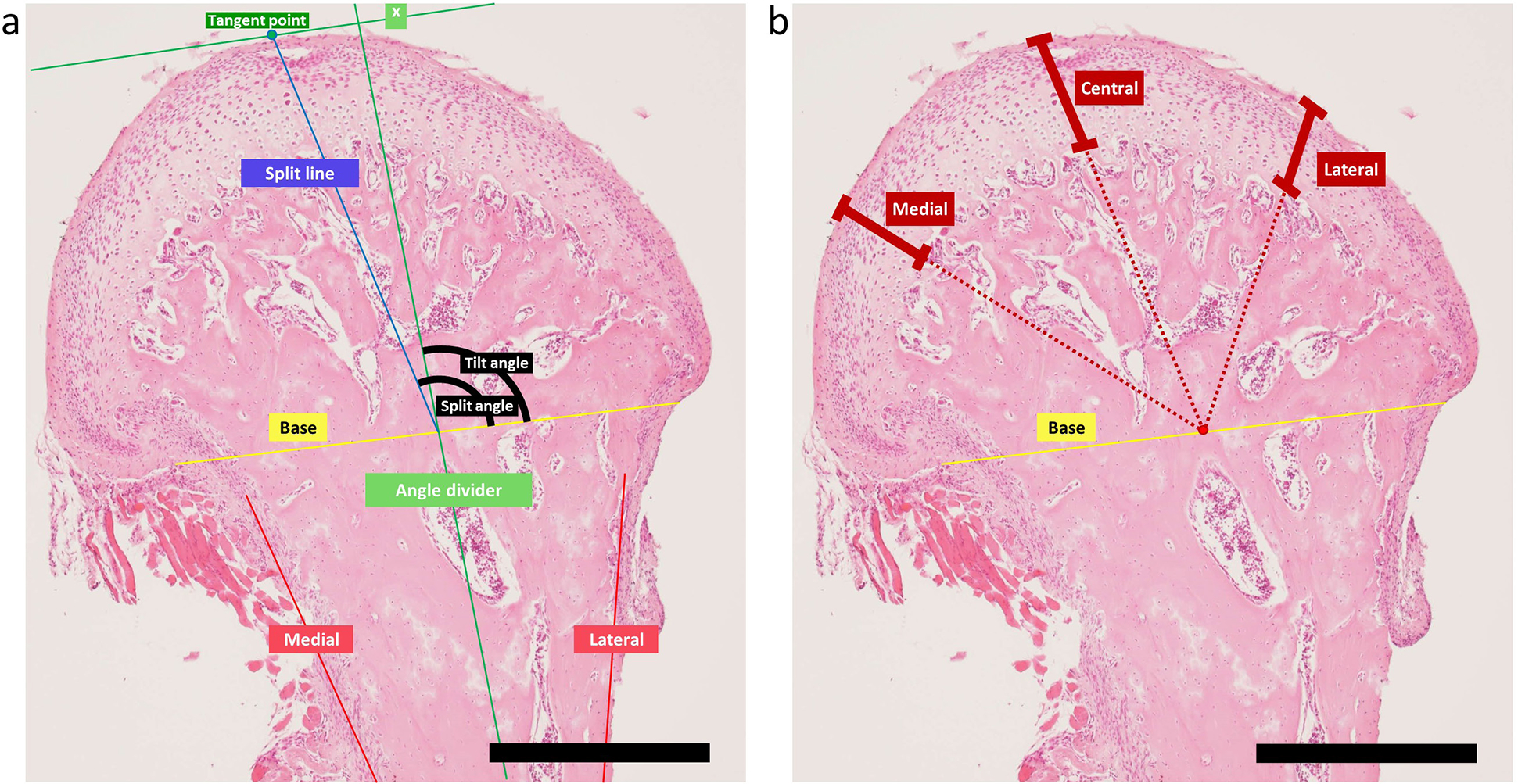
Example of landmarks used in quantitative measurements. Certain landmarks were used to determine the split and tilt angles and the thickness at the medial, central, and lateral regions. (**a**) First, the medial and lateral borders of the ramus were marked, of which the angle divider was determined. Then, the base line was drawn by connecting the medial and lateral convex points below the contour of the condyle. After, a line (x) was drawn parallel to the base line and tangential to the surface of the condyle. The split line was determined by connecting the tangent point and the midpoint of the base line. Finally, the split and base lines dictated the split angle, while the angle divider and the base line dictated the tilt angle. (**b**) In order to measure the thickness at different regions of the condyle, central line was drawn from the split line. Then the lateral and medial line were determined by dividing the angles between the central line and the base line on the medial and lateral aspect respectively. Cartilage thickness of the medial, central, and lateral regions were measured. Scale bar: 500 μm.

**Fig. 6.**
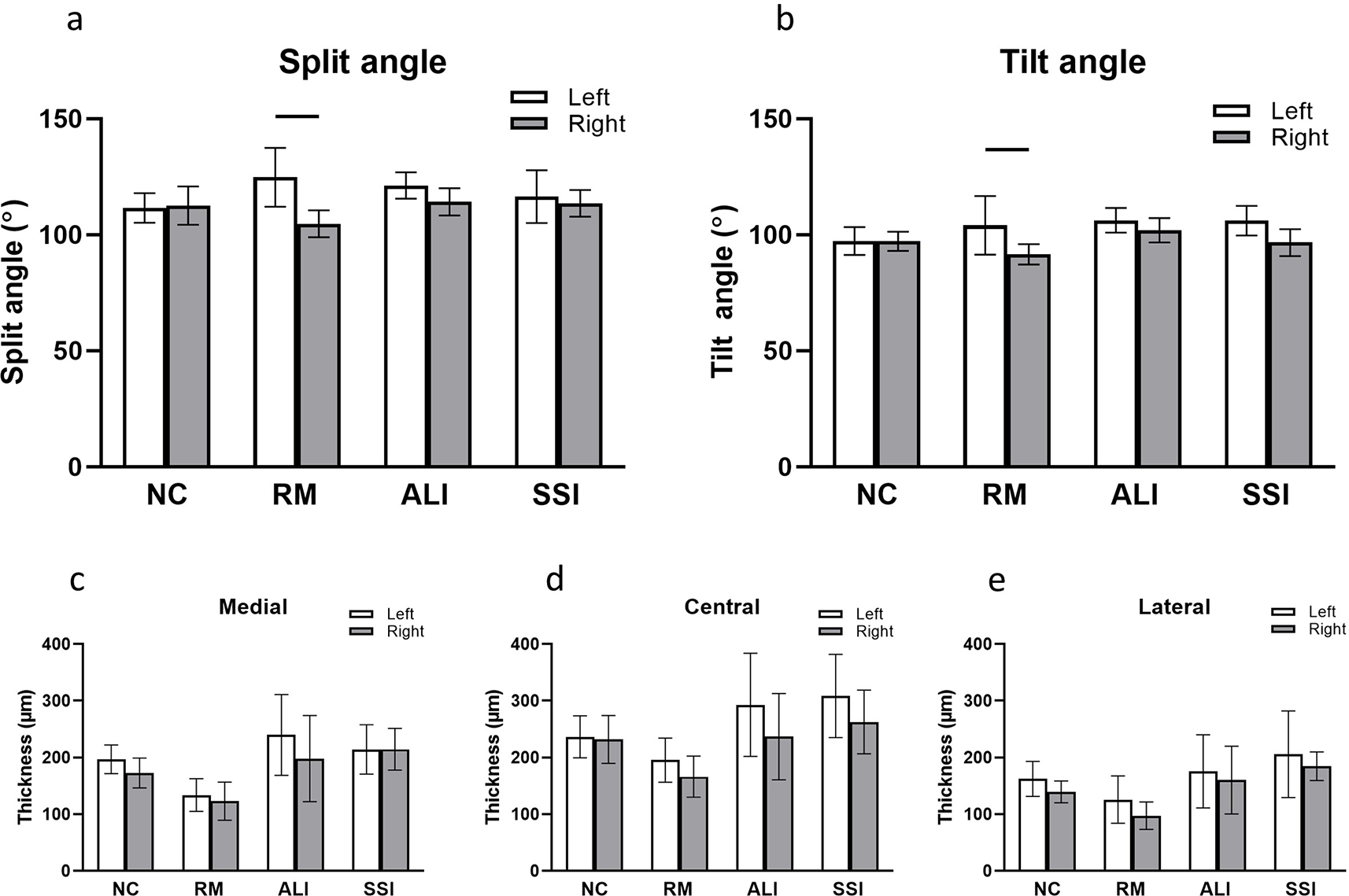
Quantitative outcomes of the angle and thickness measurements. (**a**) Split angle. (**b**) Tilt angle. Cartilage thickness at medial (**c**), central (**d**), and lateral (**e**) regions. The result was reported as mean ± standard deviation. Comparisons were made between the left and right condyles within the same group, as well as the same side between the NC group and other groups. The only significance was seen between left and right condyles of the RM group in terms of the split and tilt angles. *p* < 0.05. NC: naïve control group; RM: resin molar group; ALI: aluminum incisor group; SSI: stainless steel incisor group.

Measurements were performed with respect to landmark features that enabled the quantification of the morphological changes in the H&E images (Fig. 5). The split angle was defined as the angle between the split line and the base line (Fig. 5a). This split angle was significantly larger in the left condylar process in the RM splinted rats compared to the right condylar process in this group (124.8 ± 12.7° and 104.7 ± 5.8°, respectively. *p* < 0.05) (Fig. 6a). No other significant differences were seen in other groups when comparing the left and right side within the same group or when comparing the same side between the NC group and other groups (Fig. 6a). The changes in tilt angle, defined as the angle between the angle divider and the base line (Fig. 6b), were largely comparable to those of split angle for both splint type and side (left > right), where the only significance observed was between the left and right condylar process of the RM group (104.1 ± 12.7° and 91.6 ± 4.4°, respectively. *p* < 0.05) (Fig. 6b). The thickness of the cartilage layer at the medial, central, and lateral regions of the condylar process was also measured. However, no significant difference was detected between the left and right condylar processes within the same group, nor between the NC group and other groups when comparing the same side (Fig. 6c,d,e).

## Discussion

The impact of different types of bite-altering splints on the temporomandibular condylar process cartilage was studied. The results suggested that the RM splint is the most consistent in increasing the split angle across all three bite-altering devices, which is observed as a slant of the lateral aspect of the condylar process. Furthermore, this slanting was more drastic with the RM splints when compared to the other splinting methods, which also had a higher variability of outcomes. However, the incisor splints still showed marked tooth remodeling when compared to naïve. It is important to note that there was minimal evidence of remodeling in the fibrocartilage layers, especially when compared to the change in shape of the bone (condylar process). This suggests that the fibrocartilage layer is less sensitive to a change in abnormal loading than the bone of the condylar process. Most condylar processes from splinted animals had a change in overall shape, but all still had fibrocartilage tissue.

An unexpected finding was that the naïve tissue had a large variability in the distribution of the GAG-rich layer and condylar process shape in these 12-week-old Sprague Dawley rats. It has been reported that some species (*e.g.*, domestic dogs and wolves) have multiple variations in the shape of the condylar process even within the same breed (Curth *et al.*, 2017). Although there is no study regarding the shape variation of the condylar process in rats, the shape difference seen in the naïve group could be attributed to the morphological variation of the condylar process in rats, which makes it extremely difficult to determine early signs of OA as there is no robust GAG-rich layer in naïve animals, and the thickness throughout the condylar process is also highly variable. Thus, histological scoring systems for OA, like OARSI (Bannuru *et al.*, 2019), would not be able to detect a difference between joints from naïve and splinted rats.

To achieve the jaw shifting condition, different splinting devices were tried, where differences were observed. Due to the low strength of the flowable resin material, a cylindrical metal pin was added to each resin splint to improve wear-resistance. By unilaterally placing the resin splints on the left upper and lower molar teeth, it was possible to create an unbalanced occlusion, which resulted in the perturbation of the jaw movement upon mastication and the likely shift of the load on the TMJs. This is further verified by the slanted upper and lower incisor teeth edges in some of the RM splinted rats (Fig. 2). Previous studies have shown that the left and right condylar processes are different from each other in the unilateral splint models or functional lateral shift models (Kure-Hattori *et al.*, 2012; Sato *et al.*, 2006; Wattanachai *et al.*, 2009). In the current study, based on the results from the gross appearance, most of the changes were observed in the condylar process ipsilateral to the splint, manifested by the flattening of the lateral region.

In an attempt to generate a similar load-shifting effect on the TMJs with the incisor splints to that with the RM splint group, the incisor splints were angled in a manner similar to what was observed on the incisal edges in the RM group (the left upper incisor tooth was shorter than the right). The aluminum splint was used first, which turned out to be easily ground down by the rats. In this study, the aluminum splints were ground down differently between rats, which might contribute to the high variation in terms of how left and right joints remodeled. Nevertheless, despite this variability, the aluminum splint was still able to create an observable amount of condylar process remodeling in at least half of the animals. The joints remodeled in a different manner compared to the RM group, probably owing to the fact that the aluminum splints used were thin anterior-posteriorly, so that the lower incisor teeth moved outside the occlusion plane of the splint during mastication and the incisor teeth were pushed protrusively or intrusively by the splints, similar to the setup in other studies (Farias-Neto *et al.*, 2012; Sá *et al.*, 2017). This could be why there was a minimal amount of remodeling observed in two of the rats with aluminum splints, at least at the site analyzed, if the changes were primarily manifest in the anterior/posterior region. These would have been difficult to detect using coronal sections that show the medial-lateral view. While changes to the condylar process were not consistent, the constant grinding could have produced damage to the mastication muscles. Thus, this could be a model for myofascial pain and is worth exploring in the future.

To address the problems of the aluminum splints, SSI splints were used. They were fabricated with the same incisor teeth edge angulation as the aluminum splints, but with a much thicker anterior-posterior dimension. The SSI splint maintained its integrity throughout the entire study. However, we did not see a major change on the fibrocartilage of the condylar processes. Instead, the stainless-steel splints induced an overall change in the shape of the condylar process, likely due to bone resorption. There did appear to be more GAG staining in some condylar processes from this group compared to naïve rats, perhaps indicating a transition to hypertrophic and mineralized cartilage, but longer time points would need to be studied to assess this possibility.

Notably, the splints placed on the molar teeth might have a different compensation mechanism compared to the ones placed on the incisor teeth. The incisor teeth of rodents grow continuously, while their molar teeth do not. Therefore, when the splints were placed on the upper incisor teeth, the lower incisor teeth were grinding against the metal splint while the upper incisor teeth kept elongating. As a comparison, the incisor teeth on the molar splint rats were out of occlusion at first, but as they continue to grow, the upper and lower incisors will eventually occlude and grind against each other. The variation in the compensation of the occlusion between splint types can partially explain the outcome seen in the gross appearance and histology, where the molar splint induced a flattened area on the lateral surface of the condylar process, but the condylar process appeared to be arched in the incisor splint group.

Others have also shown that the growth-rate of the cartilage and the number of chondrocytes increased significantly after applying bilateral molar splints to rats (Buchner, 1982). Unilateral molar splints were also shown to increase the expression of aggrecan in the condylar cartilage and versican related proteoglycan in the disc and articular surface of the condylar process (Mao *et al.*, 1998). Discrepancies between the growth or ablation of the GAG layer are likely due to differences in the splinting technique and splinting material used. It is important to note that the timepoint of 4 weeks of splinting is fairly short when compared to knee OA rat models where the timepoints are usually 12–16 weeks post-injury (Miller and Malfait, 2017; Miller *et al.*, 2015). These models also cause abnormal loading by transecting the ACL (Brown *et al.*, 2020) or the medial meniscus (Allen *et al.*, 2012). For these models, transection of the medial meniscus allows for faster articulation of incongruent surfaces (femur on condyle), which produces OA faster than ACL transection models. The current TMJ splint model still has an intact TMJ disc protecting the condylar process from the fossa; therefore, this model would not be expected to develop late stage/chronic arthritis such as in knee OA models. It is, therefore, worth exploring if similarly long timepoints post splinting (12–16 weeks) produce more extensive damage to the fibrocartilage. Nevertheless, profound changes in shape of the mandibular condylar process were still found in just 4 weeks post-splinting. The impact of an animal’s sex on these changes as a result of splinting were not explored in depth, but in a few pilot male animals no differences in the slanting/skewness of the lateral aspect from splinting were observed when compared to the females at this 4-week timepoint. A further study of these sex-related differences will be needed as it is known that female rodents take much longer than males to develop OA in traumatic knee rodent models (Ma *et al.*, 2007; von Loga *et al.*, 2020).

**Fig. 7.**
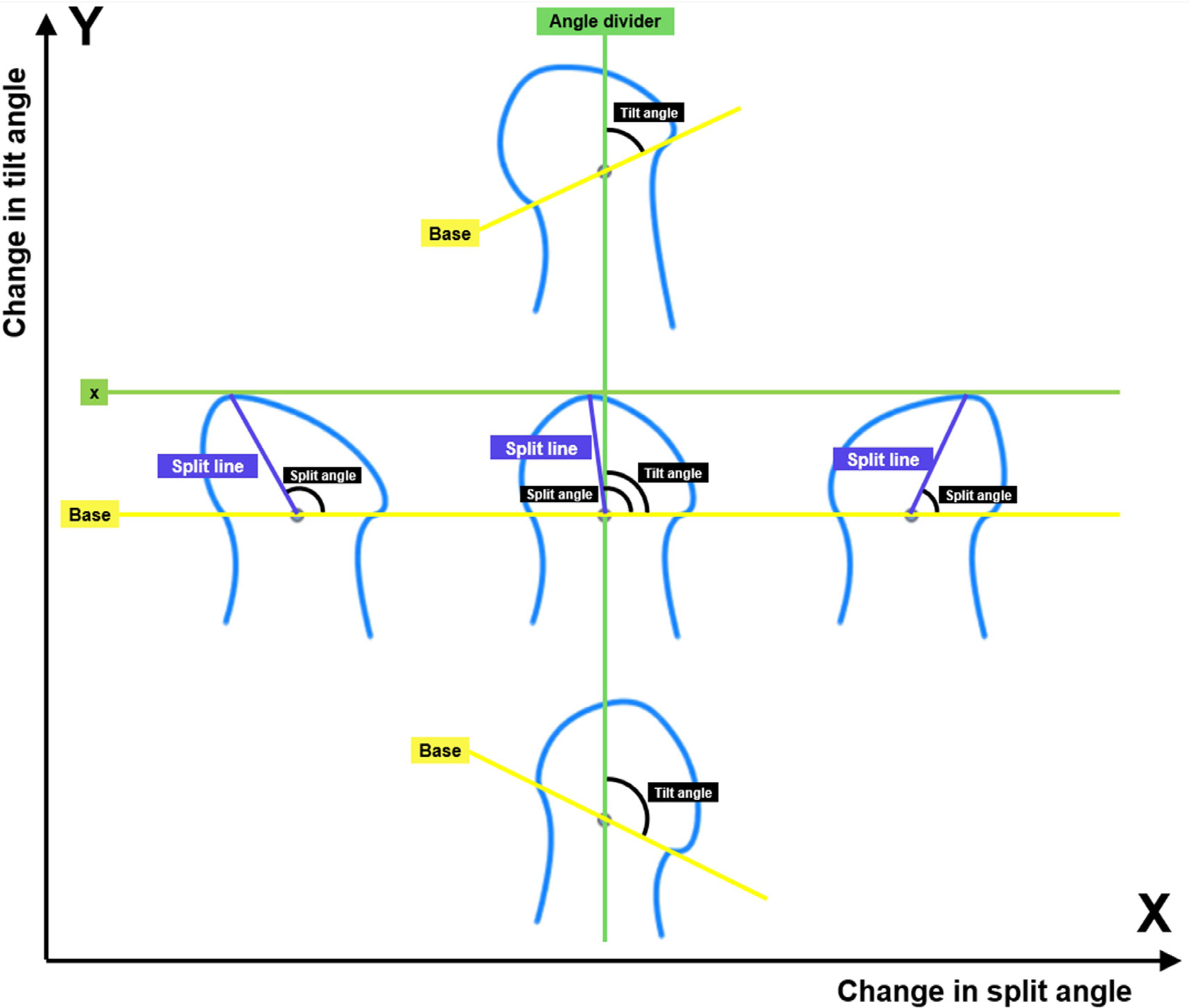
Schematic of the changes of split and tilt angles. Changes in the split angle (x-axis) reflect the changes in the outline of the condylar process (skewness). Changes in the tilt angle (y-axis) reflect the changes in the base of the condylar process relative to the ramus of the mandible (rotation).

To quantify the remodeling of the condylar process, previous studies used a line connecting the lateral and medial most-contour points as a reference to divide the condylar process into medial, middle, and lateral regions (Guo *et al.*, 2020; Sato *et al.*, 2006). However, their method only involves measurements of the area of changes in different regions that does not enable comparisons between groups. Two parameters were developed in the current study, namely the split and tilt angles, using landmark points from the H&E stained images. These two angles describe different aspects of morphological changes of the condylar processes, namely the skewness and rotation. As shown in Fig. 7, the x-axis describes changes in the split angle, during which the skewness of the condylar process outline is changed while the base of the condylar process (base line) does not rotate against the ramus (angle divider line). In contrast, the tilt angle shown on the y-axis indicates changes in the rotation of the process against the ramus, while the condylar process outline (skewness) remains the same. After performing the measurements on H&E images, the results indicated that, the remodeling for the RM group mainly happened on the left condylar processes, where the condylar process not only skewed laterally, but also rotated clockwise when comparing the left to the right. This indicated that the abnormal loading induced by the molar splint was applied to the latter side of the condylar process, which agrees with the findings from the gross appearance and histology that the lateral aspect of the ipsilateral condylar process was flattened. Contrarily, no significant difference was detected in the ALI splint and SSI splint group. In preliminary studies, it was verified that the variability of the sectioning angle did not impact on the detectable skewness or rotation of the condylar process in the coronal direction. There is the limitation in that this is a description of a 3D change in 2D, and a nuanced change could be missing that would be seen in CT imaging. However, this 2D approach still yielded meaningful and repeatable comparisons for these splinting models in the coronal plane.

The changes to the skewness and rotation of the condylar process would not be possible without the remodeling of the subchondral bone. Therefore, the split and tilt angles can serve as indicators for subchondral bone remodeling. However, μ-CT or other similar systems are still needed to precisely measure the histomorphometric changes to the bony structure, which is one of the limitations of this study. Using μ-CT would also enable the comparison of changes to the shape of the condylar process before and after the splint placement within the same individual, eliminating the influence of aforementioned morphological variations of the condylar process seen even in the naïve rats. It is important to note that μ-CT would also show subchondral bone changes, which is a key feature of DJD and its absence is a limitation of this study.

Besides the split and tilt angle, the thickness of the cartilage layer was also measured at the medial, central, and lateral regions of the condylar process. Although in some rats, the thickness in certain regions seemed to be greater, no significant difference was observed neither when comparing the left with the right side within the same group, nor when comparing the same side between the NC group and other groups. This is probably due to the large variability within each group. Therefore, a larger sample size is indicated in order to detect any significant difference, if there is any, in terms of the cartilage thickness.

A limitation of this study was that there was no comparison to a sham placement of the splints. A few sham splint placements were performed, but this was not pursued as a control method since no changes in the lateral aspect of the condylar processes were observed; therefore, saving the unnecessary use of animals. While more shams could have yielded changes in morphology, this was considered unlikely. First, all three splinting methods caused a side-to-side difference in the condylar process remodeling; while the operation, *i.e.*, mouth opening, alone affected the condylar processes bilaterally. Second, studies have reported condylar process remodeling induced by mouth opening only after a prolonged period repetitively over a few days (Kawai *et al.*, 2008; Nicoll *et al.*, 2010), one of which even shows a trend of recovery after 28 d (Kawai *et al.*, 2008). In contrast, the procedure in the current study is a one-time procedure that lasts for less than 15 min. Therefore, the impact of the procedure is estimated to be minimum after 28 d. And third, histology studies of naïve rats can give us more information on what a normal condylar process looks like, allowing the comparison of various outcomes caused by different splints. Another limitation of this study lies in the lack of the assessment of the discomfort or hypersensitivity caused by the bite-altering splints. Some rats in this study showed signs of discomfort, manifested by the skin rashes on their lower chin, which was also observed in another study using bilateral molar splints (Long *et al.*, 2019). Therefore, it is believed that the bite-altering splints can not only cause the remodeling of the condylar process, but also discomfort or even induce hypersensitivity in the TMJ region. It has also been reported that bite-altering splints are responsible for the stress-induced stimulation of the hypothalamic-pituitary-adrenal axis leading to neurological alterations predisposing the animal to hypersensitivity to stressful stimuli (Piancino *et al.*, 2019). Another limitation was that male rats were not assessed, and a comparison with females still needs to be explored.

In the current study, the splints were placed for 4 weeks, which is a relatively short time considering that it will take much longer to remodel the fibrous layer of the condylar process as shown in the histology. Therefore, studies are needed to investigate the effect of the splint on the condylar process in a longer term. Moreover, it is still unclear whether the condylar process will recover after the removal of the splint, and if it does not, knowing the timepoint when the damage becomes irreversible is of clinical significance. Therefore, future studies should also be conducted to investigate the recovery of the condylar process following the fitting of bite-altering splints. In addition to the changes to the condylar process, it has been reported that the glenoid fossa is remodeled in a rat molar splint (resin + metal crown) model (Liu *et al.*, 2007). In the current study, changes happened to the glenoid fossa were not investigated, but it would be worthwhile to study the remodeling of the glenoid fossa together with the condylar process to gain a better understanding of how the splint will affect the structure and function of the TMJ as a whole. Due to the limited scope of this study, the neuroinflammatory aspect of the painful DJD was not investigated. Therefore, future studies will be required to study the nociceptive changes of the rats and its potential link to the histological changes. Moreover, according to other studies, bite-altering splints can cause other changes to the craniofacial region, such as the morphological changes to the mandible (Mavropoulos *et al.*, 2004; Sugiyama *et al.*, 1999), changes to the periodontal tissue surrounding the splinted teeth (Liu *et al.*, 2015; Mavropoulos *et al.*, 2005), and the inflammation of the masseter (Wu and Liu, 2019), which are worth exploring – especially the inflammation of the masticating muscles as the occlusal perturbation can cause its hyperalgesia in rats (Cao *et al.*, 2009) and TMD pain is sometimes attributed to the myofascial pain (Cairns, 2010).

In conclusion, all three splints were associated with remodeling of the condylar process. However, the molar resin and SSI splints were associated with the most consistent changes in histology. It would, therefore, be most useful if future studies could be designed to assess the impact of variables such as age and sex, as well as therapeutic interventions.

## List of Abbreviations

AAALAC: Association for assessment and accreditation of laboratory animal care
ACL: anterior cruciate ligament
ALI: aluminum incisor
ANOVA: analysis of variance
CT: computed tomography
DJD: degenerative joint disease
GAG: glycosaminoglycans
H&E: hematoxylin and eosin
HSD: honestly significant difference
MCC: mandibular condylar cartilage
NC: naïve control
NIH: National Institutes of Health
OA: osteoarthritis
OARSI: Osteoarthritis research society international
RM: resin molar
SSI: stainless-steel incisor
TMJ: temporomandibular joint
UV: ultraviolet
2D: 2-dimensional
3D: 3-dimensional
μ-CT: micro computed tomography
